# Optimization of the Performance of Zirconia-Toughened and Mixed-Alumina Ceramic Tools in CNC Turning of 42CrMo4 Steel

**DOI:** 10.3390/ma19194208

**Published:** 2026-10-02

**Authors:** Irina Aleksandrova, Hristo Metev, Hristian Mitev, Trayan Hristov, Simona Tsaneva

**Affiliations:** 1 Department of Industrial Technologies and Design, Technical University of Gabrovo, 5300 Gabrovo, Bulgaria; hmetev@tugab.bg (H.M.); hmitev@tugab.bg (H.M.); trayanhristov@yahoo.com (T.H.); simona_tsaneva@abv.bg (S.T.); 2 Center of Competence “Smart Mechatronic, Eco- and Energy-Saving Systems and Technologies”, Technical University of Gabrovo, 5300 Gabrovo, Bulgaria

**Keywords:** zirconia-toughened and mixed-alumina ceramic tools, tool lifetime, roughness, multi-purpose optimization, 42CrMo4 steel, generalized utility function, Pareto-optimal solutions

## Abstract

**Highlights:**

- An approach is proposed for the multi-criteria optimization of the performance of zirconia-toughened and mixed-alumina ceramic tools under CNC turning conditions of 42CrMo4 steel
- Empirical models have been developed for the tool lifetime of zirconia-toughened and mixed-alumina ceramic tools and for the surface roughness of the machined surfaces, as a function of cutting speed and the hardness of the machined material.
- Optimal conditions have been determined for CNC turning of 42CrMo4 steel with varying hardness using zirconia-toughened and mixed-alumina ceramic tools, which provide the best combination of minimum roughness of the machined surfaces and maximum tool lifetime.

**Abstract:**

This article presents the results of a comprehensive study of the performance of zirconia-toughened and mixed-alumina ceramic tools in CNC turning of 42CrMo4 steel with hardnesses of 35, 40, and 45 HRC. Empirical models have been developed for the cutting tool lifetime and the surface roughness of the machined surfaces as a function of cutting speed and hardness of the machined material. Through multi-objective compromise optimization via two approaches (the optimal compromise region and the generalized arithmetic mean utility function with weighting coefficients) and the application of a genetic algorithm, the optimal conditions for performing the CNC turning process were determined when using tools made of different mineral ceramics for steel with varying hardness. These conditions provide a tool lifetime exceeding 8 min and a high machined surface quality with a roughness below 1.7 µm.

## 1. Introduction

Mineral ceramics are used as tool materials in various fields of engineering due to their high wear resistance, hardness, heat resistance, and low tendency to adhesively interact with the workpieces, as well as their ability to maintain their strength over a wide temperature range (up to 1000 °C). However, since they have certain drawbacks in addition to their many advantages (brittleness and high sensitivity to sudden temperature fluctuations and mechanical stress), the literature contains conflicting information regarding the areas of effective application for cutting mineral ceramics and the optimal conditions for their use [1,2,3,4,5]. Each manufacturer of mineral-ceramic inserts recommends different cutting parameters depending on the hardness and strength of the material being machined [6,7,8,9]. Furthermore, information regarding wear and force relationships, as well as the quality of machined surfaces when cutting with mineral-ceramic tools, is insufficient. All of this necessitates that each specific brand of mineral-ceramic cutting tool be used only after investigating the performance of the tools made from it and determining the optimal conditions for their operation.

The performance of mineral-ceramic cutting tools has been studied by a number of authors [10,11,12,13,14,15,16,17,18,19,20,21,22,23,24,25,26,27,28,29,30], with the lifetime/wear of the cutting tools, the surface roughness of the machined surfaces, chip formation, cutting forces, power, and temperature used as optimization parameters. Their optimal values were obtained for various combinations of process control factors (cutting speed, feed rate, cutting depth, cutting tool geometry, and hardness of the machined material).

In [10], a study was conducted on surface quality during high-speed machining of AISI 4340 steel using yttrium-stabilized zirconia-toughened alumina (ZTA) mineral-ceramic cutting inserts. Response surface methodology (RSM) was used to investigate the influence of the cutting parameters (cutting speed *v_c_*, feed rate *f*, and cutting depth *a_p_*) on the surface roughness of the machined surfaces. It was found that high-speed machining with this tool ensures good surface finish quality.

The influence of cutting speed *v_c_* and feed rate *f* on the wear/lifespan of uncoated mineral-ceramic inserts and on the surface roughness of machined surfaces during dry turning of AISI 304 stainless steel was investigated in [17]. It was found that both parameters of the cutting conditions have a significant effect on the quality of the machined surfaces and the tool life. Increasing the cutting speed leads to a deterioration in surface quality under the conditions studied. Using an accepted criterion for tool life—average wear on the rear surface *VB* = 0.1 mm—tool lives of 15 min and 9 min were achieved at cutting speeds of *v_c_* = 120 m/min and *v_c_* = 150 m/min, respectively. At lower cutting speeds, abrasive and adhesive wear mechanisms predominate, while at higher cutting speeds, diffusion wear predominates.

Aouici et al. [18] conducted a comparative study of the performance of two types of tools made from CC650 composite mineral ceramic (Al_2_O_3_ + TiC) and CC670 reinforced mineral ceramic (Al_2_O_3_ + SiC) when machining 42CrMo4 steel with a hardness of 52 HRC, without the use of a coolant. The study aims to determine the effect of cutting conditions on the roughness of the machined surfaces, as evaluated by the *Ra* criterion. The authors conducted a full factorial experiment to predict surface roughness. They investigated three levels of cutting speed *v_c_* (90, 150, 200 m/min) and feed rate *f* (0.08, 0.12, 0.16 mm/rev). The cutting depth *a_p_* ranged from 0.2 to 0.8 mm. The results show that when using the composite mineral ceramic (CC650), significantly lower values of surface roughness *Ra* (between 0.35 and 0.81 μm) were achieved. When machining with the reinforced mineral ceramic CC670, the roughness of the machined surfaces was significantly higher, ranging from 0.49 μm to 1.14 μm. Cutting forces decrease significantly with increasing feed rate and cutting speed due to the thermal softening of the steel. It has been found that when using the CC650 composite mineral ceramic, the specific force is lower, which is due to the lower coefficient of friction between the cutting tool and the workpiece. Microscopic analysis has shown that the main cause of increased surface roughness during machining with the CC670 reinforced mineral ceramic is abrasive wear on the rear surface and the formation of microcracks in the SiC structure.

Das et al. [19] applied the Taguchi method to investigate the effect of cutting speed, feed rate, and cutting depth on surface roughness when machining 42CrMo4 steel with a hardness of 45 HRC, using mineral-ceramic inserts (Al_2_O_3_ + TiCN) with a PVD-deposited titanium nitride coating. The experiments were conducted under dry cutting conditions; surface roughness was evaluated according to three criteria (*Ra*, *Rq*, *Rz*), wear was measured on the rear surface, and chip morphology was analyzed using a scanning electron microscope. It was found that cutting speed affects surface roughness; as cutting speed increased within the studied range (from 70 to 210 m/min), surface roughness decreased by approximately 4.1%. Through a variance analysis, the authors demonstrated that feed rate has the greatest influence on the change in *Ra* roughness; as the feed rate increased from 0.05 to 0.15 mm/rev, roughness increased by 84.82%.

Pal et al. [20] used composite mineral-ceramic tools (Al_2_O_3_ + TiC) when machining 42CrMo4 steel with different hardness levels (35, 45, 55 HRC) to determine the effect of the workpiece’s hardness on cutting forces, contact zone temperature, and the roughness of the machined surfaces. It was proven that surface roughness decreases as the material hardness increases. At a material hardness of 55 HRC, the lowest roughness values (*Ra* = 0.45 μm) were achieved. It was also found that the temperature in the contact zone between the tool and the workpiece increases significantly as the hardness of the workpiece material increases. At a cutting speed of 110 m/min and a workpiece hardness of 55 HRC, the temperature was 15–20% higher than that at a material hardness of 35 HRC, reaching values above 740–750 °C.

The performance of cutting tools made of a composite mineral-ceramic material (Al_2_O_3_/TiC in a 70/30 ratio) when machining Hadfield-type austenitic steel with a composition of 1.0–1.2% C and 11–14% Mn, which exhibits high impact toughness and the ability to increase its hardness under mechanical loading, was studied by Jenn-Tsong et al. [21]. The authors used RSM for experimental design and analysis. Mathematical models were developed to predict the maximum wear on the rear surface of the cutting tool and the surface roughness based on the arithmetic mean profile deviation *Ra*. The cutting speed, feed rate, radius at the cutting tool tip, and cutting time were selected as the controlling factors of the process. The experimental results showed that cutting speed is the dominant factor in terms of tool life and wear. It was found that at high speeds, abrasive wear accelerates significantly due to the high hardness of manganese steel. A larger tip radius contributes to better heat dissipation, which slightly slows down wear under heavy-duty conditions. Using RSM, the authors determined an optimal combination of control factors that led to a 9.25% reduction in wear and an 8.74% improvement in surface roughness. The lowest surface roughness was achieved via the combination of a low feed rate and a larger radius at the tip of the cutting tool. It was also proven that as cutting time increases, roughness deteriorates in direct proportion to the increase in wear. The authors demonstrated that, despite the strain hardening of Hadfield steel, the composite mineral ceramic can be successfully used for its machining by controlling the cutting speed.

In [22,23,24,25,26,27,28], studies were conducted on the performance of mineral-ceramic inserts with different tip radii—the “wiper” type—during the turning of hardened AISI D2 and AISI 4340 steels with a hardness of 60 HRC. It was found that these inserts perform better in hard turning of hardened steel in terms of roughness of the machined surfaces and tool wear compared to conventional mineral-ceramic inserts [27,28]. They allow for an increase in feed rate while ensuring a surface roughness comparable to that achieved when machining with inserts with a constant tip radius. With “wiper” inserts, a surface roughness of *Ra* < 0.8 μm was achieved [22,23]. In [26], the influence of workpiece hardness on surface roughness, cutting forces, and the wear of “wiper” inserts was investigated. Optimal cutting conditions were determined through multi-objective optimization. The experimental results confirm that the workpiece hardness has a significant effect on the surface of the machined surfaces, cutting forces, and tool wear.

Comparative studies of the tool life of mineral-ceramic and cubic boron nitride (CBN) tools when machining hardened alloy steels, using the Taguchi method, were conducted in [29]. It was found that cutting speed has a dominant influence on tool life. Optimal cutting conditions were determined, and the results show that CBN cutting tools perform better than mineral-ceramic tools.

The performance characteristics of cerium-reinforced Al_2_O_3_ mineral-ceramic inserts (CTA) during the machining of C30 steel were investigated in [30]. It was found that CTA inserts exhibit better wear resistance and surface finish quality compared to Al_2_O_3_ mineral-ceramic inserts, but they are inferior in these respects to composite mineral-ceramic (Al_2_O_3_/TiCN) inserts.

Aslantas et al. [31] conducted studies on wear, tool life, and surface finish quality during the turning of hardened AISI 52100 steel (63 HRC) using Al_2_O_3_/TiCN composite mineral-ceramic cutting inserts with and without TiN coatings. According to the results, failure modes such as fracture or chipping occur more frequently in uncoated tools, while wear on the front surface of the insert is more common in TiN-coated inserts. It was found that chip formation in coated inserts creates better thermal conditions, ensuring greater insert strength and higher quality of the machined surfaces.

Reference [32] presents the results of a comparative experimental study on the performance of Al_2_O_3_/TiC mixed-alumina tools (AT33) synthesized using microwave technology, Al_2_O_3_/TiC ceramic tools (LT55) produced by hot-pressing technology, and WC/Co cemented carbide tools (YS8) during machining of hardened 40Cr alloy steel (50 ± 2 HRC) and hardened T10A tool steel (60 ± 2 HRC). The authors found that AT33 exhibited a significantly higher tool life compared to LT55 and YS8 when machining hardened 40Cr steel at various cutting speeds. When machining hardened T10A steel, AT33 demonstrated better cutting performance compared to LT55 at lower cutting speeds (69 m/min, 104 m/min).

Comparative studies of the performance of composite mineral-ceramic inserts, reinforced mineral ceramics with silicon carbide crystal fibers, and polycrystalline cubic boron nitride (PCBN) during continuous turning of hardened 100Cr6 bearing steel (62–64 HRC) were conducted by Benga et al. [33]. The influence of cutting conditions on tool life and the quality of the machined surfaces was analyzed. Comparable surface roughness was achieved on the machined surfaces, with *Ra* values up to 0.25 μm. PCBN tools showed the best results in terms of tool life, followed by reinforced mineral-ceramic tools at low feed rates and composite mineral-ceramic tools at high feed rates.

Despite the large number of studies on the performance of mineral-ceramic tools, these studies pertain to specific machined materials and cutting conditions, primarily when turning hardened steels with a hardness exceeding 45 HRC. There is a lack of studies on the performance and effective application of ZTA and mixed-alumina ceramic tools when turning steels with a hardness below 45 HRC on CNC machines, as evaluated by the criteria of cutting tool life and surface roughness of the machined surfaces.

When turning hardened steels, mineral-ceramic cutting inserts are subjected to extreme cutting conditions due to the higher hardness of the workpiece; therefore, hardness must also be considered as one of the optimization parameters during experimental studies in order to assess its impact on tool performance under various machining conditions.

In this regard, the objective of this article is to optimize the performance of ZTA and mixed-alumina ceramic tools when finishing turning 42CrMo4 steel with hardnesses of 35, 40, and 45 HRC on CNC machines. This was carried out in the following sequence: investigation and modeling of the influence of cutting speed and hardness of the machined material on tool performance, based on the criteria of cutting tool life and surface roughness of the machined surfaces, and multi-objective compromise optimization of the performance of the studied mineral-ceramic inserts and determination of Pareto-optimal solutions through the application of a genetic algorithm.

The principal scientific novelty of the study is as follows:

- Evaluation and comparison of the overall behavior of two different classes of ceramics (Al_2_O_3_ + ZrO_2_ and Al_2_O_3_ + TiCN) during the finishing turning of 42CrMo4 steels with a hardness of less than 45 HRC, which has direct practical value for the industry;
- Optimalization of the performance of these ceramic tools in terms of tool lifetime and surface roughness of the machined surfaces to meet the requirements of actual production;
- A comparative analysis of the performance criteria of the tools under optimal cutting conditions, determined using the methods of the optimal compromise region and the generalized utility function with weighting coefficients.

## 2. Investigation and Modeling of the Performance of Zirconia-Toughened and Mixed-Alumina Ceramic Tools

### 2.1. Equipment, Materials, Methods

The objective of the study is to establish the correlation between the performance of ceramic tools made of ZTA and mixed-alumina ceramic—assessed based on the criteria of tool life and surface roughness of the machined surfaces, on the one hand—and the cutting conditions during CNC turning, on the other.

**Figure 1:**
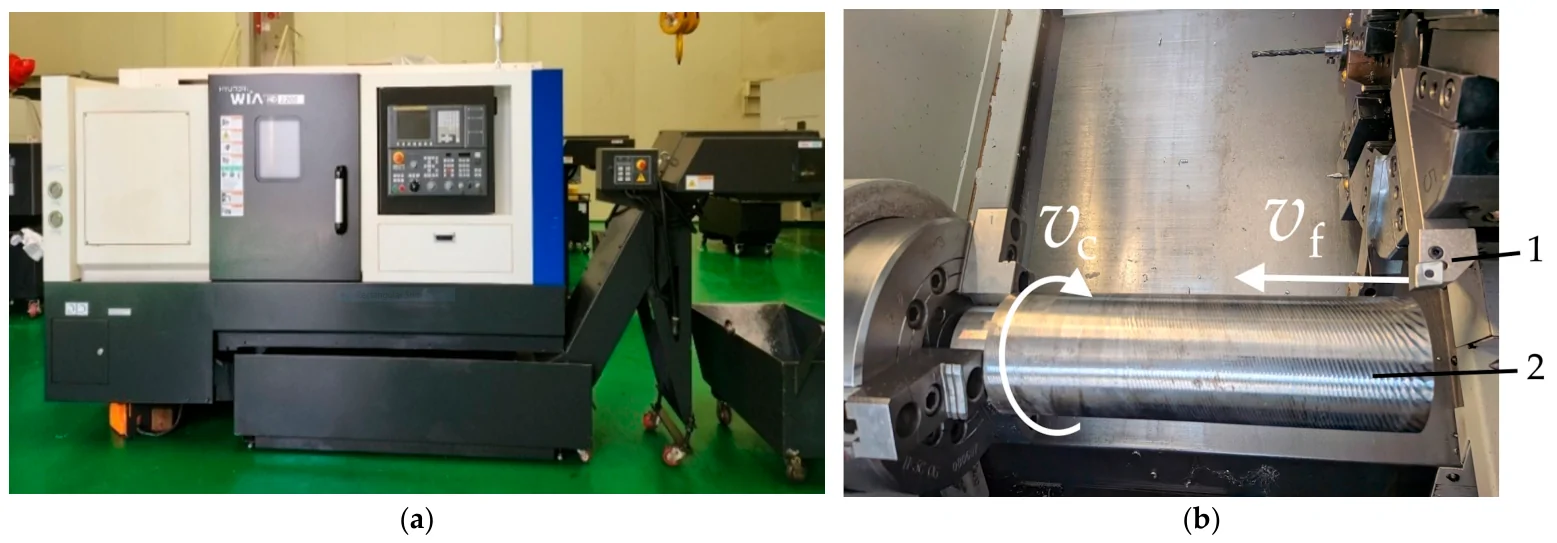
Hyundai WIA HD 2200 lathe (**a**) and work area (**b**): 1—cutting tool; 2—workpiece.

To achieve this objective, the experiments were conducted on a Hyundai WIA HD 2200 CNC lathe (Hyundai WIA Machine Tool, Changwon, South Korea) (Figure 1) while finishing machining workpieces had a diameter of *D* = 120 mm, length of *L* = 250 mm, and roughness of *Ra* = 2 µm. The material being machined was 42CrMo4 steel (EN ISO 683-2:2018 [34]); its chemical composition and physical-mechanical properties are presented in Table 1. The workpieces were heat-treated by quenching to hardnesses of 35 HRC, 40 HRC, and 45 HRC.

Turning tools with indexable ZTA and mixed-alumina ceramic inserts from the Nikko Tools [6] product range were used as cutting tools. The tool designations, their geometries, and the specific insert types used (T01 and T02) along with their substrate-coating combinations are presented in Table 2. These tool-insert combinations are evaluated as two independent experimental groups tailored to their respective chemical and mechanical properties.

The experimental studies were conducted without the use of a coolant, since the ceramic tool materials are prone to thermal shocks.

The cutting speed (*v_c_*, m/min) and the Rockwell H hardness (HRC) of the workpiece were selected as the controlling factors. Their variation levels are as shown in Table 3. These values fall within the limits recommended in the literature and meet the requirements related to the service life of mineral-ceramic tools and the quality of the machined surfaces [6]. It should be noted that in actual manufacturing environments, workpiece hardness is a fixed constraint determined by design requirements and heat treatment, rather than a parameter adjusted by the machine operator. However, including hardness as a control factor in the regression models and as an optimization variable carries significant scientific and practical value; it provides mechanical and manufacturing engineers with quantitative data to balance mechanical properties against machinability during the design stage, while enhancing the model’s predictive capability to automatically adapt the cutting speed to the specific hardness of the incoming material. Taking into account the decisive influence of cutting speed and the hardness of the workpiece on the tool lifetime of ceramic cutting tools and the quality of the machined surfaces, the experimental studies were conducted at a constant cutting depth of *a_p_* = 1.5 mm and a constant feed rate of *f* = 0.2 mm/rev.

Based on preliminary information, it has been assumed that the general form of the models describing the correlation between the performance criteria (tool life *T_i_* (min) and roughness of the machined surfaces *Ra_i_* (µm)) of the studied ZTA and mixed-alumina ceramic tools and the group of independent variables—with the cutting speed *v_c_* (*X*_1_) and hardness of the machined material *H* (*X*_2_) as control factors—is

(1) $$Y_{\mathrm{pi}}=B_{0}+B_{1}X_{1}+B_{2}X_{2}+B_{11}X_{1}^{2}+B_{22}X_{2}^{2}+B_{12}X_{1}X_{2},$$

where *Y*_1*i*_ = *T_i_* and *Y*_2*i*_ = *Ra_i_* (*i* = 1,2—tool material index; *i* = 1 for ZTA ceramic inserts; *i* = 2 for mixed-alumina ceramic inserts), and *B*_0_, *B*_1_, *B*_2_, *B*_11_, *B*_22_, *B*_12_ are regression coefficients.

To construct the regression models (1), an optimal central composite design with *N* = 2*^k^* + 2.*k* + 1 = 9 trials was applied (*k* = 2—the number of control factors, whose levels in coded and natural form are presented in Table 3). The experimental design and analysis of the experimental results were carried out according to the methodology specified in [37,38], where multiple regression analysis and QStatLab version 6.1.1.3 were used [39].

Microscopic analysis has demonstrated that the primary causes of deterioration in the quality of machined surfaces when using mineral-ceramic tools include abrasive wear on the rear surface (*VB*), the formation of microcracks, chipping, and thermal defects. In the finishing machining of steel, where a minimum surface roughness (*Ra* = 1.6 − 3.2 µm) must be ensured, surface quality begins to deteriorate linearly with tool wear even in the early stages of its operation, long before its complete failure occurs as a result of the aforementioned failure mechanisms [1,2,3,4,5]. This necessitates the use of a technological criterion for tool wear—corresponding to a predefined value of the surface roughness of the machined surface—from among the known criteria.

Based on the recommendations of manufacturers of mineral-ceramic cutting tools for finishing CNC turning and requirements for the machined surface, the machining time required to achieve a surface roughness of *Ra* = 2 µm was selected as the technological wear criterion for determining tool lifetime [6,7,8,9].

The values of the performance criteria (*T_i_* and *Ra_i_*) for the studied mineral-ceramic tools in each trial were determined in the following sequence:

- Preliminary single-factor experiments were conducted to investigate the change in the roughness of the machined surfaces as a function of cutting time *t*. Machining was performed using cutting tools with ZTA and mixed-alumina ceramic inserts at cutting speeds of *v_c_*, and with material hardness *H* corresponding to the variation levels in Table 3. The roughness of the machined surface was evaluated using the parameter “arithmetic mean deviation from the profile” *Ra*. To ensure high statistical representativeness, each recorded *Ra* value was determined as the arithmetic mean of three independent measurements performed in different zones around the workpiece circumference. The experimental tests were progressively conducted until the surface roughness explicitly exceeded the value of *Ra* > 3 µm. The measurements were performed using a Mitutoyo SJ-201 profilometer [40]. The instrument’s operation was in accordance with ISO 3274:1998 [41] through utilization of an inductive probing system outfitted with a diamond stylus tip (tip radius *r* = 2 µm, 60° angle) and a standard contact force of 0.75 mN. Measurement and evaluation of parameter *Ra*, which covers values below and above 2 µm, were performed in accordance with EN ISO 4287:1997 [42]. The instrument has a high resolution of 0.001 μm within a Z-axis measuring range of ±200 μm. Depending on the measured *Ra* range, standard cut-off lengths (λ*_s_*) of 0.8 mm (for *Ra* ≤ 2 µm) and 2.5 mm (for *Ra* > 2 µm) were used during testing to ensure reliable computation of the parameter in accordance with ISO recommendations.
- Single-factor regression models were constructed for the machined surface roughness as a function of cutting time *t* (s), the general form of which is:

*Ra_ijl_* = *A*_0_ + *A*_1_*t* + *A*_2_*t*^2^ + *A*_3_*t*^3^, (2)

where *j* is the workpiece hardness index (*j* = 1 for 35 HRC; *j* = 2 for 40 HRC; *j* = 3 for 45 HRC); *l* is the cutting speed index (*l* = 1 for *v_c_* = 250 m/min; *l* = 2 for *v_c_* = 300 m/min; *l* = 3 for *v_c_* = 350 m/min); and *A*_0_, *A*_1_, *A*_2_, *A*_3_ are regression coefficients. The obtained results were analyzed using QstatLab software, version 6.1.1.3 [39].

- The performance criteria for the studied mineral-ceramic tools were determined based on the obtained regression models (2) (Table 4) as follows: the surface roughness of the machined surface after a machining time *t* = 300 s (5 min), and the tool lifetime of the cutting tools as the machining time required to reach a specified technological criterion—roughness of the machined surface at *Ra* = 2 µm. In this study, the primary focus is placed on establishing the technological tool life, as surface quality represents the critical limiting factor in precision finish turning operations. Operationally, a cutting tool is considered to have reached the end of its service life the moment it violates the surface roughness requirements, regardless of the physical wear state. While this approach effectively predicts the operational lifecycle from a workpiece quality perspective, the physical failure mechanisms of the ceramic insert (such as chipping, microcracking, and brittle fracture) fall outside the scope of the constructed models, and will be the subject of future research.

This two-step approach serves as an engineering method for data standardization, establishing a balanced dataset required for subsequent second-level RSM regression. By minimizing the impact of random experimental error, this mathematical filtering ensures high input data stability and exceptional approximation accuracy (high coefficient of determination) in the secondary response models.

### 2.2. Results and Modeling

The experimental design and data on the performance criteria of the tools under study are presented in Table 5. The values for the operational lifetimes of the cutting tools made of ZTA and mixed-alumina (*T*_1_ and *T*_2_) and the roughness of the surfaces machined with them (*Ra*_1_ and *Ra*_2_) were determined in accordance with the methodology presented in Section 2.1; the single-regression models (2), along with the statistical analysis of the experimental results, are shown in Table 4. The single-factor models include only the significant regression coefficients, defined according to the condition $\hat{t}>t_{\alpha /2,\nu }$, where $\hat{t}$ and *t*_α/2,_ are, respectively, the calculated value of Student’s *t*-test for each of the regression coefficients and the tabulated value of Student’s *t*-test (α = 0.05—significance level; ν = *n* − *m*—number of degrees of freedom; *n*—number of observations; *m*—number of coefficients in the model). The regression models are adequate ($\hat{F}>F_{\alpha ,\nu_{1}}{{,}_{\nu }}_{2}$; $\hat{F}$ and $F_{\alpha ,\nu_{1},\nu_{2}}$—calculated and tabulated values of Fisher’s criterion) with a 95% confidence level, and the relationships between the roughness of the machined surfaces and the cutting time are described with a high level of accuracy. The values of the coefficients of determination are ${\hat{R}}^{2}=0.5486-0.9873$.

The results concerning tool lifetime and the roughness of the machined surfaces, given in Table 5, were analyzed using QstatLab software [39]. The resulting models and the statistical analysis of the experimental results are presented in Table 6, while the corresponding analysis of variance (ANOVA) parameters are provided in Table 7. The statistical analysis of the experimental results includes determination of the regression coefficients of model (1) and testing their importance using Student’s *t*-test, and verification of the regression model’s adequacy by calculating the coefficient of determination and verifying its significance using Fisher’s *F*-test.

The regression models (Table 6), consider only the significant regression coefficients, determined by the condition $\hat{t}>t_{\alpha /2,}$, where $\hat{t}$ and *t*_α/2,_ are, respectively, the calculated value of Student’s *t*-test for each of the regression coefficients and the tabulated value of Student’s *t*-test (α = 0.05—significance level; ν = *N* − *m*—number of degrees of freedom; *N*—number of trials; *m*—number of coefficients in the model) (Table 6).

The models are adequate, since they satisfy the condition $\hat{F}>F_{\alpha ,\nu_{1},\nu_{2}}$ with a 95% confidence level, and accurately describe the relationships between the performance criteria and the controlling factors. The calculated values of $\hat{F}$ and the tabulated values of $F_{\alpha ,\nu_{1},\nu_{2}}$ for Fisher’s criterion for each of the regression models are given in Table 6. The values of the adjusted coefficients of determination are ${\hat{R}}_{\mathrm{adj}}^{2}=0.9764-0.9998$. For every regression model, the values of the predicted *R*-squared (${\hat{R}}_{\mathrm{pred}}^{2}=0.9343-0.9997$) were determined (Table 6). These values indicate that the models are capable of accurately predicting new data. Regarding the lack-of-fit test, a formal statistical partition could not be mathematically computed due to the specific nature of the experimental design. The values for tool life and surface roughness of the components machined with white and mixed-ceramic cutting tools were determined based on the established single-factor regression models. Since the matrix of the optimal central composite design does not contain replicated trials at the experimental points, including the center point, there are no degrees of freedom available to calculate the pure error. Consequently, the statistical adequacy of the models is robustly verified via the high significance of the *F*-test, Student's *t*-test for the coefficients, and the high values of both the adjusted (${\hat{R}}_{\mathrm{adj}}^{2}$) and predicted (${\hat{R}}_{\mathrm{pred}}^{2}$) coefficients of determination (Table 6), confirming that the discrepancy between them is minimal, the residual variance is low, and the models possess exceptional predictive capability and reliability.

**Figure 2:**
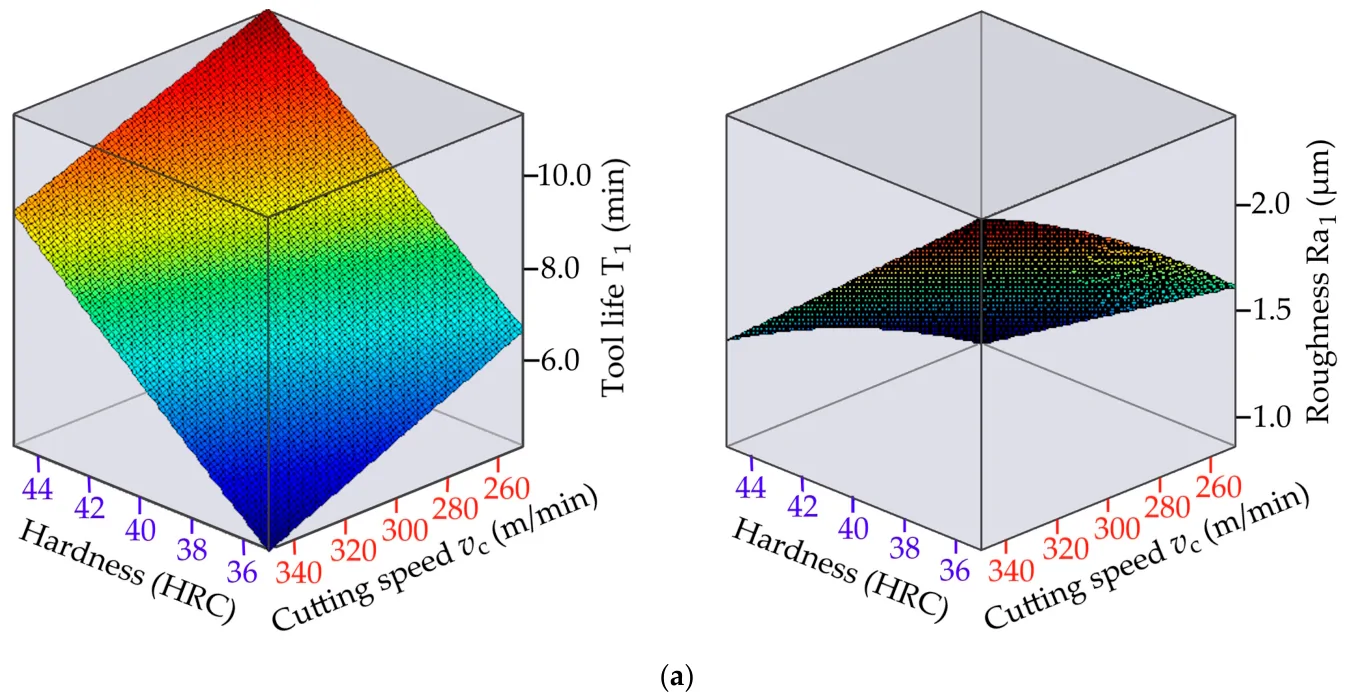

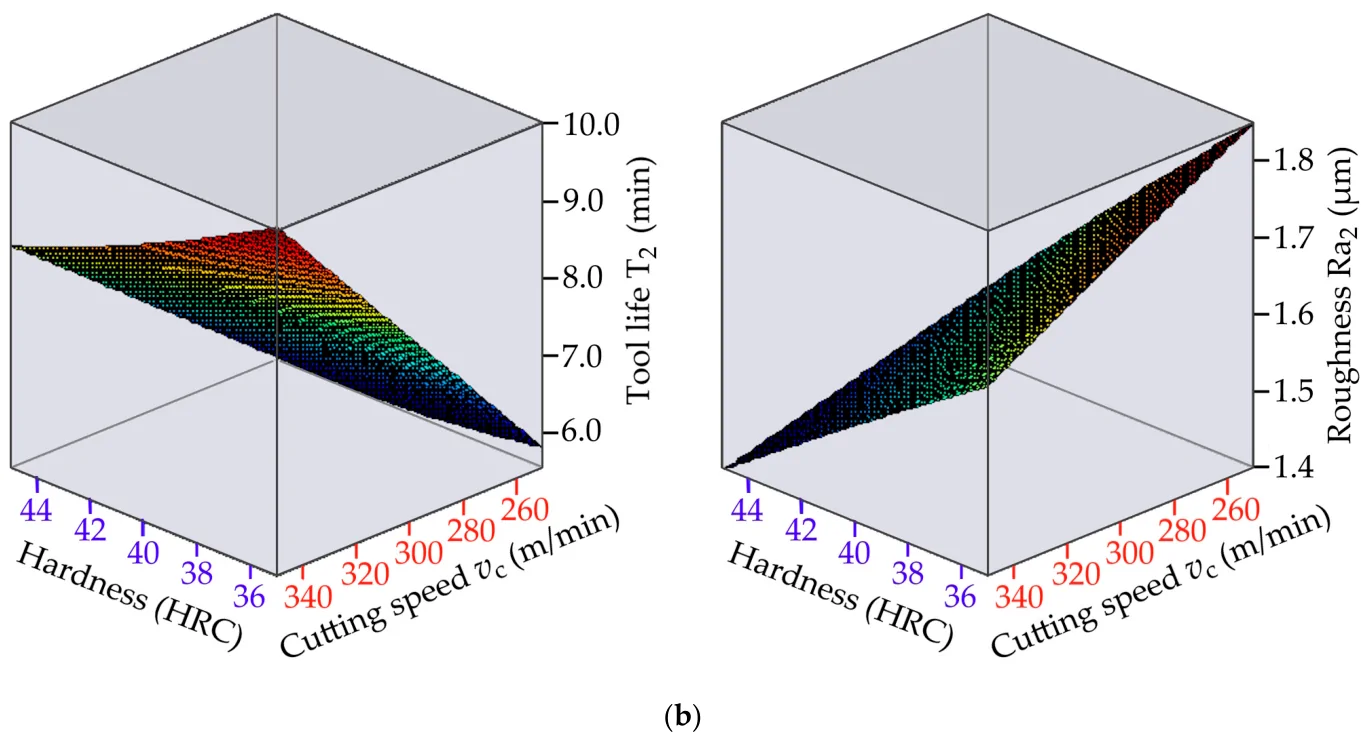
Effect of cutting conditions on the service life of cutting tools and the roughness of machined surfaces when machining with cutting tools made of: (**a**) ZTA ceramic; (**b**) mixed-alumina ceramic.

**Figure 3:**
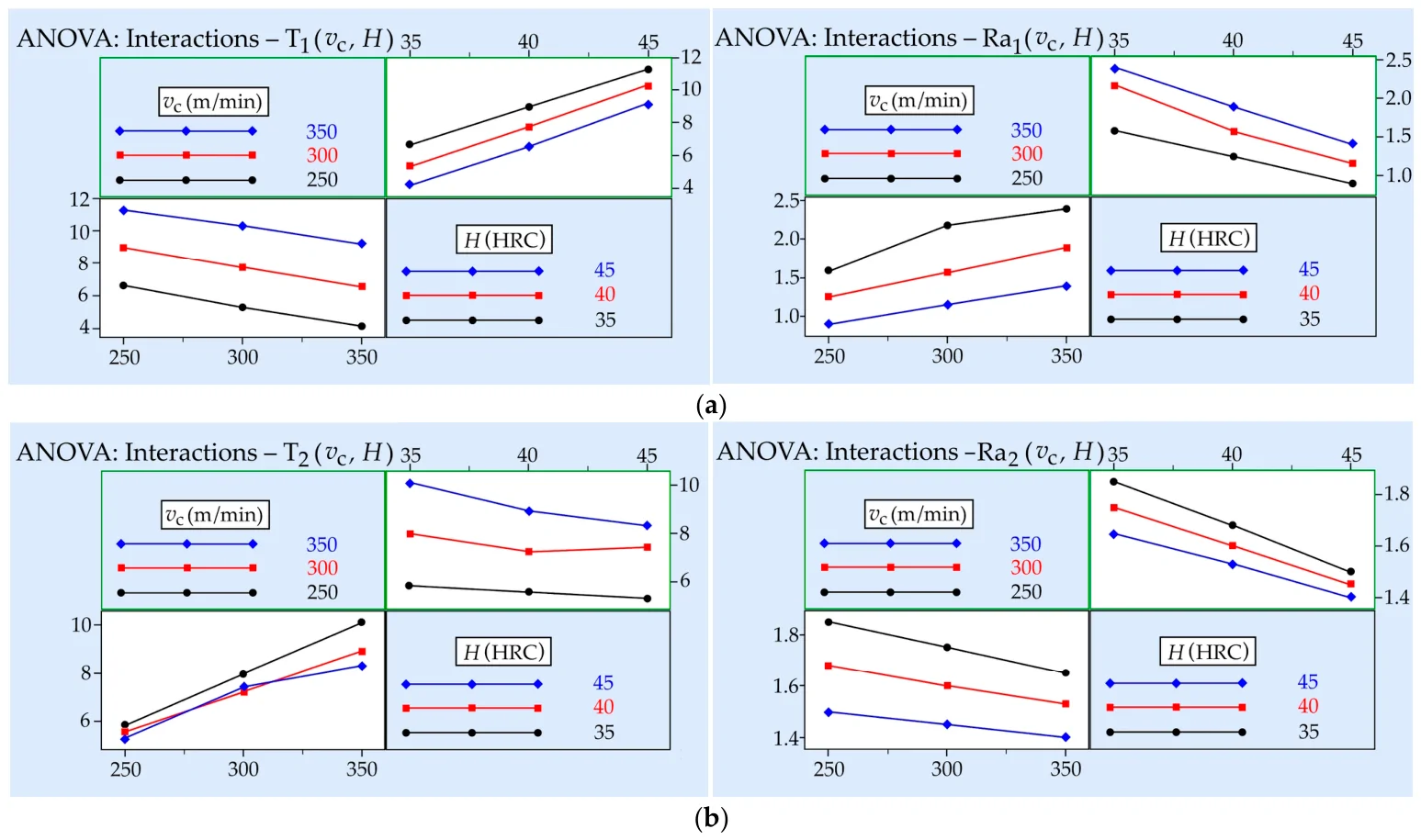
Visualization of the effect of cutting conditions on the service life of cutting tools and the roughness of machined surfaces when machining with cutting tools made of: (**a**) ZTA ceramic; (**b**) mixed-alumina ceramic.

The impact of cutting conditions on the service life of cutting tools and the roughness of the machined surfaces, in line with the regression models developed (Table 6), is presented graphically in Figure 2. An ANOVA was performed to determine the degree of influence of the controlling factors on the performance criteria under study, using the QStatLab software [39]. Table 7 provides the comprehensive ANOVA parameters (sum of squares, degrees of freedom, mean square, *F*-value, and *p*-value) structured directly against the overall model, each individual control factor, and the total variation for each of the investigated parameters, with the obtained results also graphically illustrated in Figure 3.

### 2.3. Analysis of the Results

Analysis of the obtained regression models (Table 6) and the graphical relationships plotted based on them, as well as the results of the conducted analysis of variance (Table 7, Figure 3), show that the performance criteria studied (tool life and roughness) during the machining of 42CrMo4 steel depend on the cutting conditions and the type of tool material, as follows:

- Changes in the hardness of the workpiece have a stronger effect on the tool life of ZTA ceramic tools than on the cutting speed (Table 7); as hardness increases, tool life increases by up to 48.4% (Table 8). The effect of hardness is more pronounced at higher cutting speeds. As the cutting speed increases, the tool life of ZTA ceramic tools decreases (to 26.9%), while the tool life of mixed-alumina ceramic tools increases (to 26.2%) (Table 8). For mixed-alumina ceramic tools, cutting speed has a stronger effect on tool life (Table 7), with the longest tool life achieved at a cutting speed of *v_c_* = 350 m/min and a steel hardness of 45 HRC (*T* = 8.33 min) (Table 9). As the hardness of the workpiece increases, tool life decreases by up to 7% (Table 8).
- At low cutting speeds and low hardness for the 42CrMo4 steel under study, ZTA ceramic tools produce a lower roughness of the machined surfaces (up to 1.2 times lower) compared to composite-ceramic tools. At high cutting speeds and high hardness of the machined material, the roughness decreases (to *Ra* = 1.4 μm) and is comparable when machining with both types of ceramic inserts (Table 9). The hardness of the machined material has a stronger effect on the roughness during turning with both types of mineral ceramics (Table 7). Changes in the hardness of the steel have a greater effect on the roughness when machining with ZTA ceramic tools; as hardness increases, the roughness decreases by up to 47.2%. When machining with mixed-alumina ceramic tools, an increase in hardness leads to a reduction in roughness of up to 17.14% (Table 9). As the cutting speed increases, the roughness increases when machining with ZTA ceramic tools (up to 26.2%) and decreases when machining with mixed-alumina ceramic tools (down to 19.0%) (Table 8).

The observed opposite trends in terms of tool life and surface roughness at elevated cutting speeds can be attributed to the distinct thermo-mechanical properties, material compositions, and coatings of the cutting inserts under thermal loads. As the cutting speed increases, a significant amount of heat is generated, inducing thermal softening of the workpiece and reducing cutting resistance. Although this phenomenon occurs with both tool types, their performance depends heavily on their thermal stability and resistance to abrasive and diffusion wear. For the ZTA tool, high temperatures cause thermal degradation and a loss of its transformation toughness in the zirconia phase (ZrO_2_), leading to accelerated thermal wear, plastic deformation, and microchipping of the cutting edge; this decreases tool life and increases surface roughness. Conversely, the mixed-alumina ceramic tool exhibits superior hot hardness, high thermal stability, and chemical inertness. As a result, it resists diffusion wear and chemical dissolution, maintains its cutting edge integrity, and benefits from the workpiece softening. This ensures a clean shearing action and lower cutting forces, leading to a simultaneous increase in tool life and a reduction in surface roughness [1,2,3,4,5]. While the conducted analysis proves the patterns in the variation in tool performance, the detailed wear mechanisms of the ZTA and mixed-alumina inserts under different cutting conditions are not investigated in the present article, but are an objective for future research.

The results confirm the applicability of the developed regression models for predicting the service life of cutting tools and the roughness of machined surfaces when machining 42CrMo4 steel. The developed models can be used to select optimal machining conditions in order to increase the service life of cutting inserts and improve the quality of the machined surfaces.

A comparison of the performance of ZTA ceramic and mixed-alumina ceramic cutting tools when machining 42CrMo4 steel, based on the criteria of tool life and roughness of the machined surfaces, shows that performance depends on both the cutting speed and the hardness of the machined material. This requires performing a multi-objective optimization to determine combinations of material hardness and cutting speed that ensure minimum roughness of the machined surfaces and maximum cutting tool life.

## 3. Optimization of the Performance of Cutting Tools Made of ZTA and Mixed-Alumina Ceramic

### 3.1. Methodology for Multi-Objective Optimization

To determine the CNC turning process conditions that ensure the optimal performance of ZTA and mixed-alumina ceramic tools, two multi-objective compromise optimization methods were implemented: the determination of an optimal compromise region, and multi-objective optimization using a generalized utility function with weighting coefficients—to achieve these aims, the non-dominated sorting genetic algorithm (NSGA-II) [43,44] with QStatLab version 6.1.1.3 [39] was used.

The use of this certified tool is justified by its key computational advantages [43,44]. The NSGA-II algorithm is characterized by low mathematical complexity thanks to a fast non-dominance sorting procedure, a built-in elitism strategy, and a clustering distance operator. This operator ensures diversity and uniform distribution of points along the Pareto front, preventing premature local convergence, which is critical for accurately determining the optimal compromise region. Although both the integrated NSGA-II algorithm and the newer alternative methods belong to the general class of metaheuristics, the choice of this industry-proven tool guarantees the stability of the solutions and provides an objective basis for calculating the optimal values for both methods, without the need for additional tuning of specific algorithmic parameters, which carries a high risk.

The genetic operators in the algorithm were defined based on software-implemented and industry-validated configurations set by default in QStatLab for engineering regression models: population size—50, number of iterations—2500, and number of generations—50. The “Simulated Binary Crossover” operator was used with a probability of 0.9 and a crossover distribution index of 10. The mutation operator applied was “Polynomial Mutation” with a mutation distribution index of 100 and a probability determined by the classical 1/n rule (where n is the number of variables). Since the optimization space consists of only two continuous independent variables (*v*_c_ and *H*), the mutation probability is mathematically fixed at 0.5.

The inherent risks of evolutionary algorithms, such as early stagnation or entrapment in local extrema, are entirely eliminated in this work due to the characteristics of both the algorithm and the optimization surface itself. In a two-dimensional search space governed by smooth polynomial regression models, the optimization surface inherently lacks complex multimodal local optima. Furthermore, in the multi-objective optimization mode, the crowding distance mechanism of the NSGA-II effectively preserves genetic diversity, preventing the Pareto front from collapsing into local optima. Concurrently, in the single-objective mode during the execution of the scalarized desirability function, the high crossover probability combined with the built-in elitism strategy ensures reliable and direct convergence toward the exact global maximum without any computational anomalies.

#### 3.1.1. Determination of the Optimal Compromise Region

This method necessitates the resolution of the following system of inequalities:

(3) $$\left|\begin{array}{c} T_{i}\left(X_{1}^{*},X_{2}^{*}\right)=T_{i\max}>T_{i}\left(X_{1},X_{2}\right)\\ {\mathrm{Ra}}_{i}\left(X_{1}^{*},X_{2}^{*}\right)={\mathrm{Ra}}_{I\min}>{\mathrm{Ra}}_{i}\left(X_{1},X_{2}\right)\\ T_{i}\left(X_{1}^{*},X_{2}^{*}\right)\geq \left[T\right]\\ {\mathrm{Ra}}_{i}\left(X_{1}^{*},X_{2}^{*}\right)\leq \left[\mathrm{Ra}\right]\\ X_{1\min}\leq X_{1}\leq X_{1\max}\\ X_{2\min}\leq X_{2}\leq X_{2\max} \end{array}\right.,$$

where $X_{1}^{*}=v_{c}^{*}$ (m/min) and $X_{2}^{*}=H^{*}$ (HRC) are, respectively, the optimal values for the cutting speed and the hardness of the material being machined; [*T*] and [*Ra*] are the acceptable values for the response variables under study, specified depending on the type of machining.

The values of the performance criteria *T_i_*(*X*_1_,*X*_2_) and *Ra_i_*(*X*_1_,*X*_2_) are determined in line with the regression models presented in Table 6, while the minimum and maximum values of the cutting speed and the hardness of the machined surface are established according to Table 3.

The objective comes down to determining Pareto-optimal solutions for each of the investigated tool materials that satisfy the system of inequalities (3), using the NSGA-II and QStatLab software.

#### 3.1.2. Multi-Objective Optimization Based on a Generalized Utility Function

The optimization problem is solved via the following sequence [37,38]:

1. Regression models are constructed for the generalized arithmetic mean utility function, with weight coefficients depending on the control factors of the CNC turning process, the general form of which is:
  (4) $$\Phi_{\mathrm{ir}}=C_{0}+C_{1}X_{1}+C_{2}X_{2}+C_{11}X_{1}^{2}+C_{22}X_{2}^{2}+C_{12}X_{1}X_{2},$$
  where *i* = 1,2 is the tool material index, and *r* is the index corresponding to the method of determining the Φ*_ir_* depending on the objective of the study (*r* = 1 when the objective is to ensure a combination of maximum cutting tool life and minimum roughness of the machined surfaces; *r* = 2 when the priority is to ensure maximum service life of the cutting tools; and *r* = 3 when the priority is to ensure minimum roughness of the machined surfaces).

The models are constructed based on multifactorial experiments designed to investigate the impact of the control factors on the generalized utility function, performed according to an orthogonal central composite design.

The generalized arithmetic mean utility function with weighting coefficients is determined for each experiment according to the following relationship:

Φ*_ir_* = *w_T_*η*_Ti_* + *w_Ra_*η*_Rai_*, (5)

where η*_Ti_*, η*_Rai_* are utility coefficients, and *w_T_*, *w_Ra_* are weight coefficients for the response variables under study.

The utility coefficients are determined by the following relationships:

η*_Ti_* = *k_T_*(*T_i_* − *T_u_*)/(*T*_max_ − *T*_min_), (6)

η*_Rai_* = *k_Ra_*(*Ra_i_* − *Ra_u_*)/(*Ra*_max_ − *Ra*_min_), (7)

where *T_i_* and *Ra_i_* are determined in accordance with the regression models presented in Table 6 for each combination of control factors according to the experimental design; *T*_max_, *T*_min_, *Ra*_min_, *Ra*_max_ are utility limits of the studied response variables; *T_u_*, *Ra_u_* are the least useful values of the response variables obtained within the limits of the factor space; and *k* = ±1 coefficients accounting for the utility of the increase in the respective studied performance criterion.

The values of the weighting coefficients for each of the generalized arithmetic mean utility functions are, respectively: *w_T_* = 0.5; *w_Ra_* = 0.5—for *Φ*_11_ and *Φ*_21_; *w_T_* = 0.7; *w_Ra_* = 0.3—for *Φ*_12_ and *Φ*_22_; *w_T_* = 0.3; and *w_Ra_* = 0.7 —for *Φ*_13_ and *Φ*_23_.

2. Using a genetic algorithm and the QStatLab software, combinations of values for the control factors $\left(X_{1}^{*},X_{2}^{*}\right)$ in the studied factor space are determined, for which the generalized arithmetic mean utility functions with weighting coefficients Φ*_ir_* have a maximum value for each of the cutting tools in accordance with the following conditions:
  (8) $$\left|\begin{array}{c} \Phi_{\mathrm{ir}}\left(X_{1}^{*},X_{2}^{*}\right)={\left(\Phi_{\mathrm{ir}}\right)}_{\max}\geq \Phi_{\mathrm{ir}}\left(X_{1},X_{2}\right)\\ T_{i}\left(X_{1}^{*},X_{2}^{*}\right)\geq \left[T\right]\\ {\mathrm{Ra}}_{i}\left(X_{1}^{*},X_{2}^{*}\right)\leq \left[\mathrm{Ra}\right]\\ X_{1\min}\leq X_{1}\leq X_{1\max}\\ X_{2\min}\leq X_{2}\leq X_{2\max} \end{array}\right.,$$
  where the permissible values [*T*] and [*Ra*] of the performance criteria for the studied ceramic inserts are specified depending on the type of machining.

### 3.2. Determination of Optimal Cutting Conditions Using the Optimal Compromise Region Method

The optimal combinations of cutting speed $v_{c}^{*}$ (m/min) and hardness of the machined surface *H*^∗^ (HRC) have been predicted, at which maximum tool lifetime and minimum surface roughness are achieved for each of the cutting tools (Table 10). The criteria for cutting tool lifetime and surface roughness of the machined surfaces are T ≥ 8.0 min and Ra ≤ 1.7 µm. These limits are in accordance with the recommendations of manufacturers of mineral-ceramic cutting tools, specifically from the catalogs of Nikko Tools, Seco Tools, Sandvik Coromant, and Iskar, in order to ensure both the quality of the machined surfaces and a high production rate [6,7,8,9]. They also take into account the values for the lifetime of the cutting tool and roughness determined under the projected optimal cutting conditions (Table 10).

Unlike carbide tools, mineral-ceramic tools allow for much higher cutting speeds. For this reason, a short tool lifetime is accepted in exchange for an exceptionally high production rate and good surface finish. For finishing turning of steel on CNC machines using mineral-ceramic tools, the catalogs of the manufacturers listed above recommend an economic tool lifetime in the range of 10 to 20 min. The roughness limit was selected based on the requirements for the machined surface (*Ra* = 2 µm) and take into account that, when finishing machining steel, a minimum surface roughness must be ensured on the machined surfaces (*Ra* = 1.6 − 3.2 µm).

**Figure 4:**
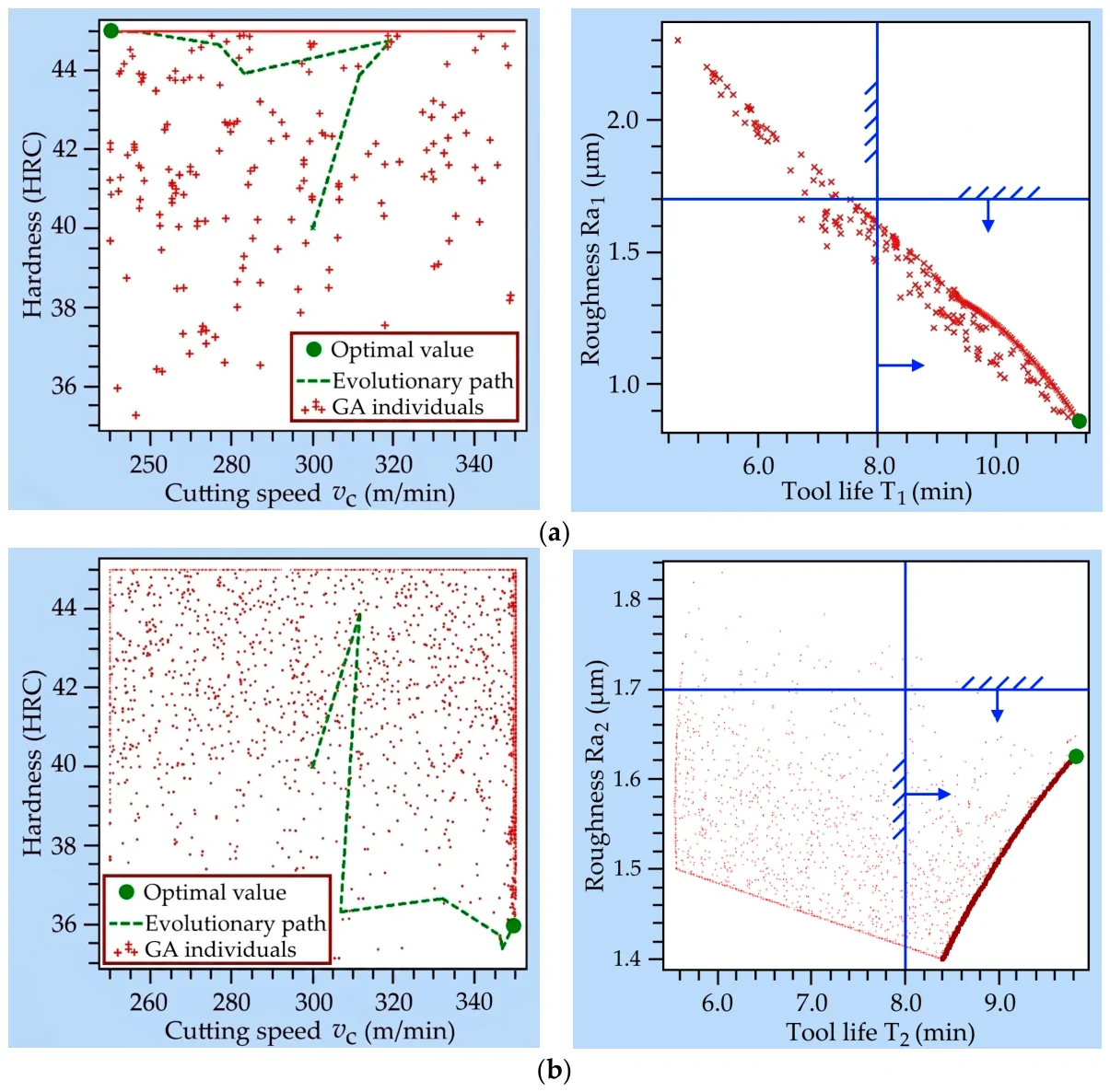
Visualization of the determination of optimal cutting conditions: (**a**) zirconia-toughened alumina ceramic tools; (**b**) mixed-alumina ceramic tools.

A visualization of the method used to find the optimal solution for each of the mineral-ceramic inserts is shown in Figure 4.

### 3.3. Multi-Objective Optimization Using the Generalized Utility Function Method

The optimization problem was solved for each of the mineral-ceramic inserts used (Table 2). The experimental design, with the values of the generalized arithmetic mean utility function determined in Equation (5), is presented in Table 11.

Following a statistical analysis of the experimental results using multivariate regression analysis and QstatLab software [39], regression models were created for the generalized arithmetic mean utility functions. These models include only the significant regression coefficients, based on the condition $\hat{t}>t_{\alpha /2,\nu }$ (Table 12). The regression models are adequate ($\hat{F}>F_{\alpha ,\nu_{1}}{{,}_{\nu }}_{2}$) with a 95% confidence level and describe, with high accuracy, the relationships between the generalized utility functions and the controlling factors. The values of the coefficients of determination are ${\hat{R}}^{2}=0.9880-0.9981\;\mathrm{and}\;{\hat{R}}_{\mathrm{pred}}^{2}=0.9442-0.9910$ (Table 12).

**Figure 5:**
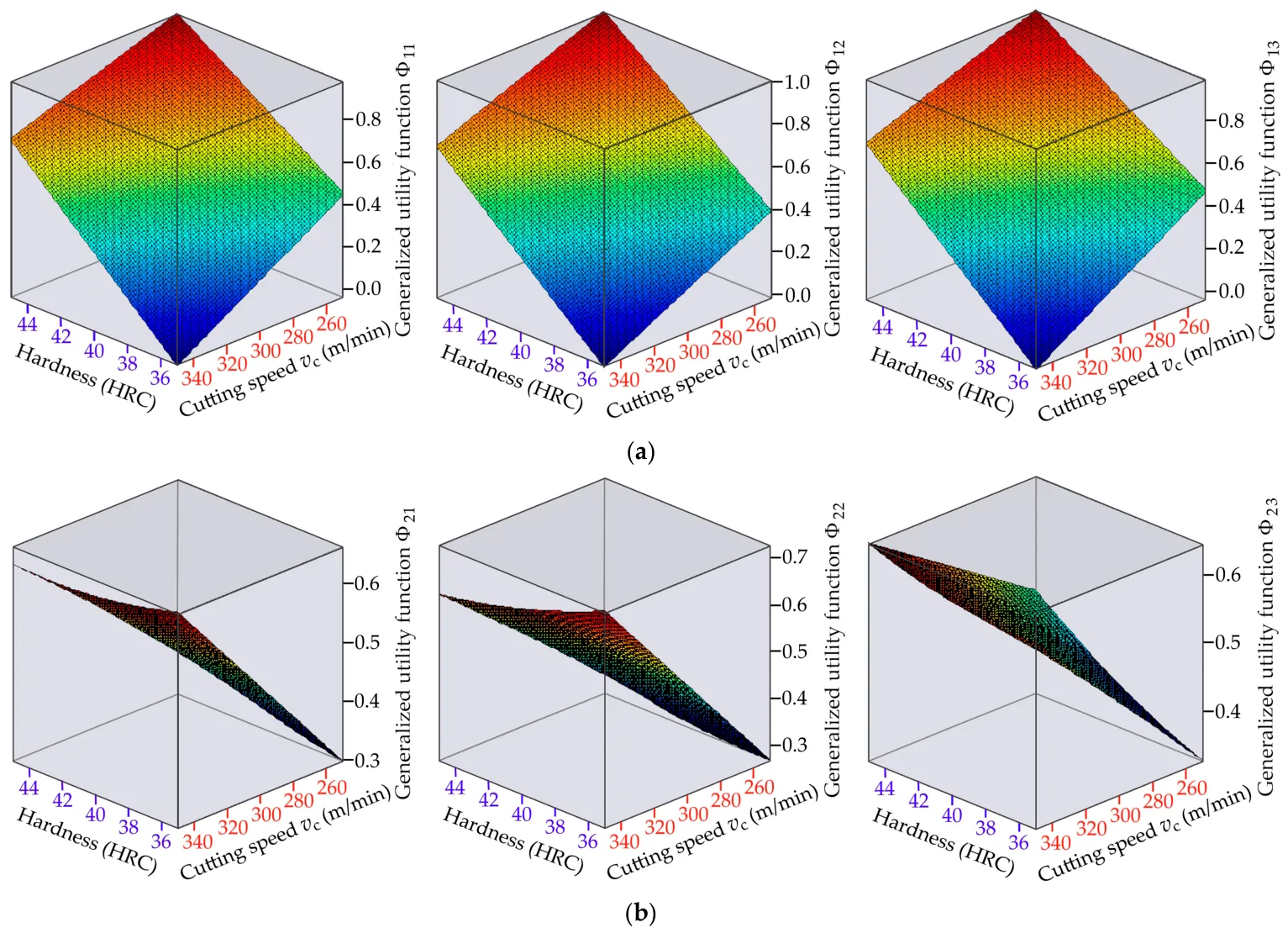
Effect of cutting conditions on the generalized utility functions: (**a**) ZTA ceramic tools; (**b**) mixed-alumina ceramic tools.

**Figure 6:**
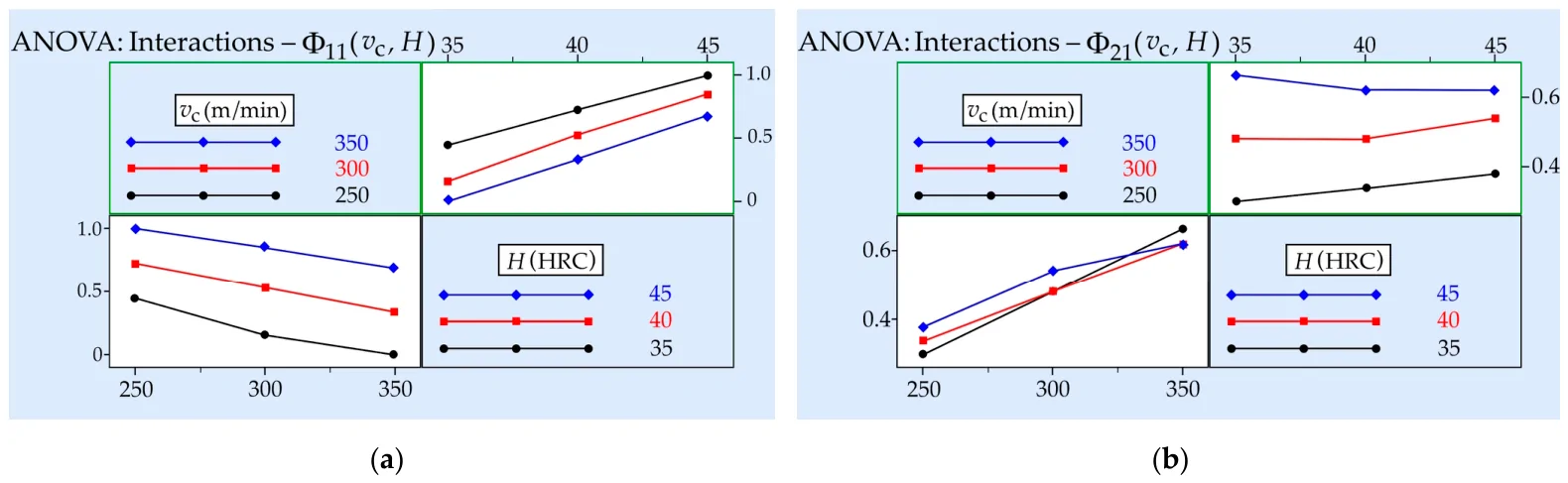
Visualization of the effect of cutting conditions on the generalized utility functions Φ_11_ and Φ_21_: (**a**) ZTA ceramic tools; (**b**) mixed-alumina ceramic tools.

The effect of cutting conditions on the generalized arithmetic mean utility functions, in compliance with the developed models, is depicted graphically in Figure 5. To determine the degree of effect of the controlling factors, an analysis of variance (ANOVA) was performed, which is presented in Figure 6 for the generalized utility functions Φ_11_ and Φ_21_.

The regression model analysis (Table 12) and the graphical relationships based on them (Figure 5), as well as the interpretation of the ANOVA results (Figure 6), show that when machining with ZTA ceramic tools, the hardness of the machined surfaces has a greater influence on the generalized arithmetic mean utility functions Φ_11_, Φ_12_, and Φ_13_. An increase in *H* within the studied range leads to an increase in Φ_11_, Φ_12_, and Φ_13_. When machining with mixed-alumina ceramic tools, the influence of cutting speed *v_c_* is greater and, as cutting speed increases, the generalized arithmetic mean utility functions Φ_21_, Φ_22_, and Φ_23_ increase. Changes in hardness do not lead to significant changes in the functions Φ_21_, Φ_22_, and Φ_23_.

The optimal combinations of cutting speed $v_{c}^{*}$ and workpiece surface hardness H^*^ have been predicted to ensure maximum values of the generalized utility functions for each of the mineral-ceramic inserts used, subject to the following constraints: *T* ≥ 8.0 min; *Ra* ≤ 1.7 µm (Table 13).

**Figure 7:**
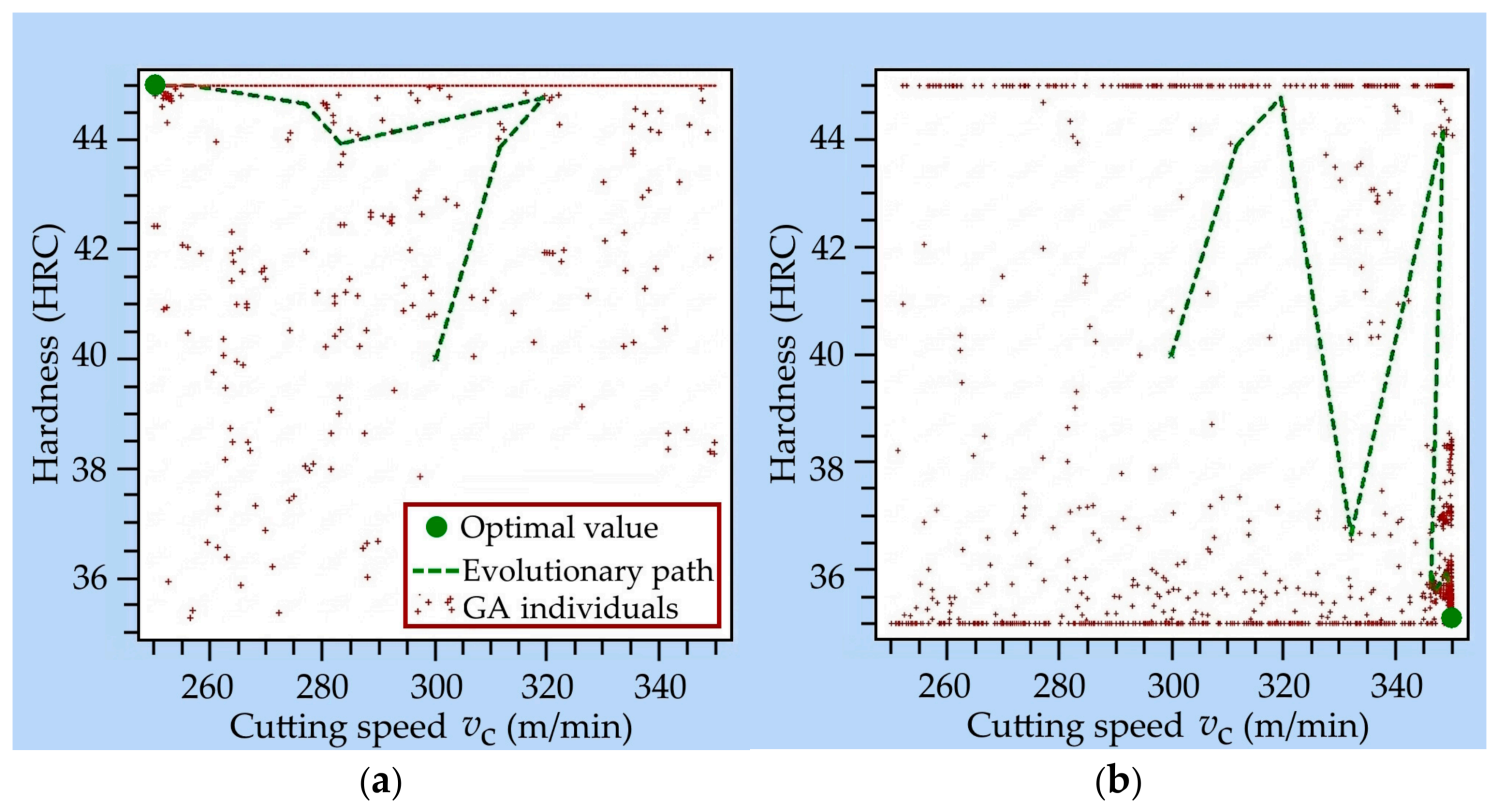
Visualization of the determination of optimal cutting conditions using the generalized utility function method, ensuring a combination of maximum tool life and minimum roughness of machined surfaces: (**a**) ZTA ceramic tools; (**b**) mixed-alumina ceramic tools.

**Figure 8:**
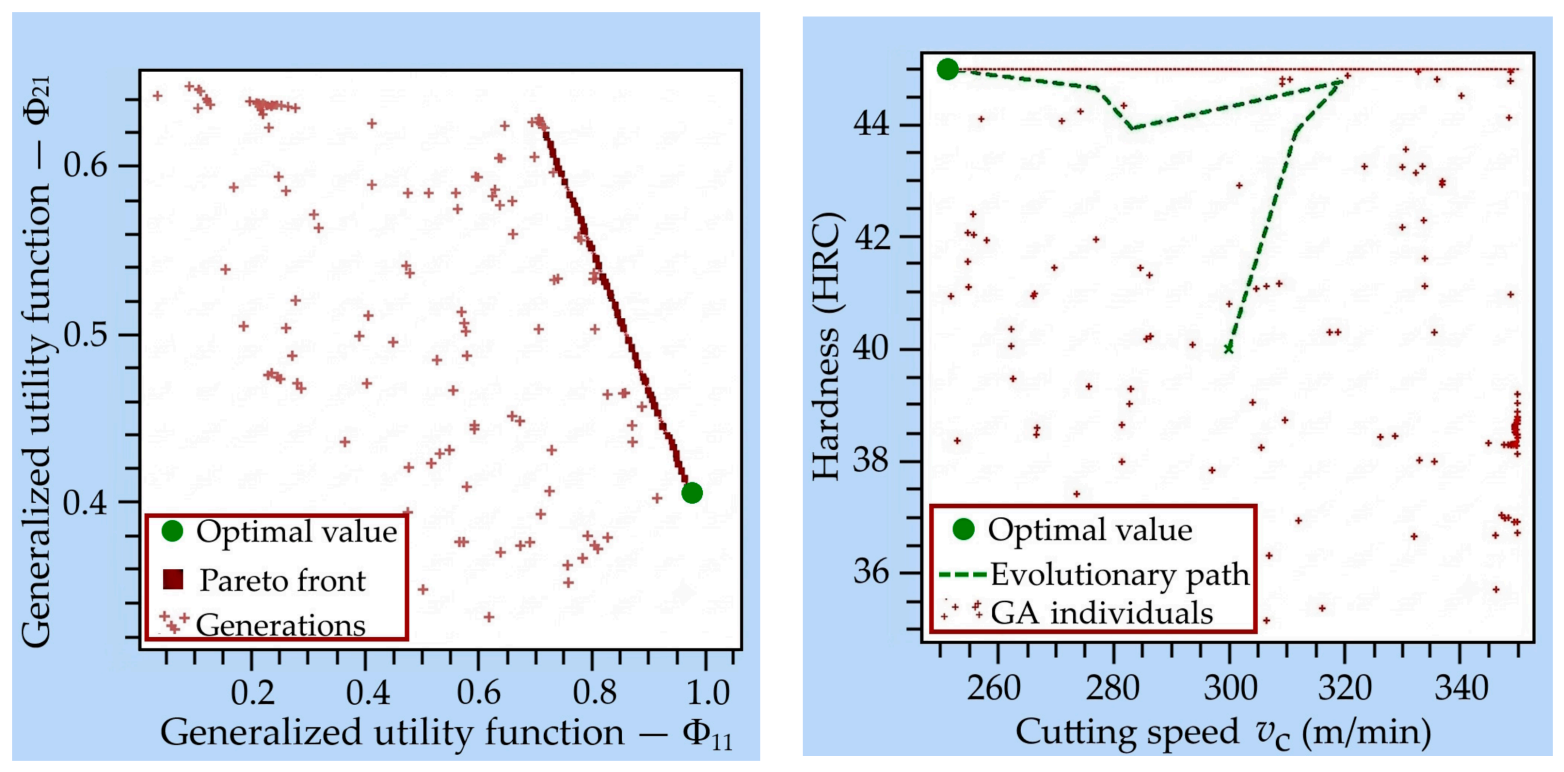
Optimal cutting conditions ensuring a combination of the maximum values of the generalized utility functions Φ_11_ and Φ_21_.

A visualization of the method for finding the optimal solution using the generalized utility function method for each of the mineral-ceramic inserts used is presented in Figure 7. The optimal cutting conditions, which ensure a combination of the maximum values of the generalized utility functions Φ_1*r*_ and Φ_2*r*_, are presented in Table 14 (Figure 8).

### 3.4. Comparative Analysis of Performance Criteria

A comparison of the performance criteria of ZTA and mixed-ceramic cutting tools in CNC turning under the optimal conditions determined by the two multi-objective optimization methods reveals the following (Table 10 and Table 13):

- Comparable tool life for ZTA ceramic tools and roughness of the machined surfaces for both optimization methods, with differences of only 0.04% and 3.6%, respectively;
- Comparable tool life for mixed-alumina ceramic tools and roughness of the machined surfaces when optimized using the optimal compromise region method and the generalized utility function method, when the goal is to simultaneously ensure maximum tool life and minimum roughness, as well as when the sole priority is to ensure maximum tool lifetime (the differences between the criteria are 2.6% and 1.4%, respectively);
- Greater tool lifetime (by 15.3%) of composite-ceramic tools when optimized using the optimal compromise region method, and lower surface roughness (by 13.9%) under the optimal conditions determined by the generalized utility function method, where the priority is to ensure minimum roughness.

## 4. Conclusions

As a result of the conducted research, modeling, and optimization of the performance of ZTA and mixed-alumina ceramic tools during CNC turning of 42CrMo4 steel with varying hardness, the following conclusions can be drawn:

- High-accuracy regression models were developed for the performance criteria (tool lifetime and surface roughness), effectively reflecting the influence of cutting speed and workpiece hardness. These models enable the selection of optimal machining conditions to improve the service life of the cutting inserts and the quality of the machined surfaces.
- It has been established that changes in workpiece hardness exhibit a stronger influence on the tool lifetime of ZTA ceramic tools than cutting speed. For tools made of mixed-alumina ceramic, the opposite trend is observed—cutting speed has a more influential effect on tool lifetime.
- Increasing the cutting speed leads to opposite trends in surface quality, causing the surface roughness to increase when machining with ZTA ceramic tools, but to decrease when machining with mixed-alumina ceramic tools. Changes in the hardness of the machined material have the most significant effect on the roughness when machining with ZTA ceramic tools.
- Using multi-objective compromise optimization based on the methods of the optimal compromise region and the generalized arithmetic mean utility function with weighting coefficients, and by applying a genetic algorithm, the optimal cutting conditions (Table 10 and Table 13) for CNC turning with ZTA and mixed-alumina ceramic tools were determined. These conditions ensure a tool life exceeding 8 min and a surface finish with a roughness of less than 1.7 µm.
- A comparative analysis of the performance criteria of the studied tools was carried out under optimal cutting conditions, as determined by the two optimization methods. Comparable results (in terms of tool lifetime and roughness of the machined surface) were observed for both ZTA and mixed-alumina ceramic tools. This correspondence holds true when the priorities in the generalized utility function method are directed toward simultaneously ensuring maximum tool life and minimum roughness, or solely toward achieving maximum tool life. When machining with mixed-alumina ceramic tools, higher tool life (by 15.3%) is achieved under the cutting conditions specified by the optimal compromise region method. Lower roughness (by 13.9%) is achieved under the optimal conditions established by the generalized utility function method, when the priority is to achieve minimum roughness.
- While the conducted analysis proves the patterns in the variation in tool performance, the detailed wear mechanisms of the ZTA and mixed-alumina inserts under different cutting conditions are not investigated in the present article, but are an objective for future research.

## Figures and Tables

**Table 1:** Chemical composition and physical-mechanical properties of 42CrMo4 steel.

Chemical Composition								Tensile Strength (MPa)	Hardness (HRC)
C (%)	Si (%)	Mn (%)	Ni (%)	Cr (%)	Mo (%)	P (%)	Cu (%)		
0.405	0.239	0.652	0.110	0.894	0.180	0.012	0.227	850	35; 40; 45

**Table 2:** Cutting tool and ceramic insert specifications.

Cutting Tool		Inserts			
Designation (ISO 5608 [35])	Geometry	Designation (ISO 1832:2017 [36])	Material	Coating	Manufacturer/Grade
NT-DCLNR2525M12X	Tool major cutting-edge angle κ*_r_* = 95° Tool minor cutting-edge angle $\kappa_{r}^{\prime }=5{}^{\circ}$ Tool orthogonal rake angle γ_0_ = −6° Tool orthogonal clearance angle α_0_ = 7° Nose radius *r*_ε_ = 0.5 mm	CNGA120408-CC (T01)	Zirconia-toughened alumina ceramic (Al_2_O_3_ 90%+ ZrO_2_ 10%)	Silver PVD coating (CrN/TiAlN)	Nikko Tools NAZ 300
		CNGA120408-CC (T02)	Mixed-alumina ceramic (Al_2_O_3_ 70% + TiCN 30%)	PVD coating (TiCN)	Nikko Tools NAC 200

**Table 3:** Levels of control factors.

Control Factors		Factor Levels		
		−1	0	+1
*X*_1_	*v_c_* (m/min)	250	300	350
*X*_2_	*H* (HRC)	35	40	45

**Table 4:** Single-factor regression models for roughness as a function of cutting time and statistical analysis.

Insert	Control Factors		Regression Models	Student’s Criterion	Fisher’s Criterion		Coefficient of Determination
	*H*	*v_c_*			$\hat{F}\geq F_{\alpha ,\nu_{1},\nu_{2}}$		
	(HRC)	(m/min)		*t*_α/2,_	$\hat{F}$	$F_{\alpha ,\nu_{1},\nu_{2}}$	${\hat{R}}^{2}$
T01	35	250	*Ra*_111_ = 1.9846 − 0.0107*t* + 4.057∙10^−5^*t*^2^ − 3∙10^−8^*t*^3^	2.4469	148.08	4.7571	0.9859
		300	*Ra*_112_ = 1.9031 − 0.2123*t* + 1.2377∙10^−3^*t*^2^ − 3∙10^−7^*t*^3^	2.4469	155.8426	4.7571	0.9873
		350	*Ra*_113_ = 1.8570 − 0.2064*t* + 1.116∙10^−4^*t*^2^ − 1.6∙10^−7^*t*^3^	2.4469	139.7547	4.7571	0.9859
	45	250	*Ra*_131_ = 2.3626 − 0.0099*t* + 2.439∙10^−5^*t*^2^ − 2∙10^−8^*t*^3^	2.4469	53.0559	4.7571	0.9637
		300	*Ra*_132_ = 2.3309 − 0.014017*t* + 4.512∙10^−5^*t*^2^ − 4∙10^−8^*t*^3^	2.4469	10.2164	4.7571	0.8363
		350	*Ra*_133_ = 2.0229 − 0.0086*t* + 2.8∙10^−5^*t*^2^ − 2∙10^−8^*t*^3^	2.4469	19.7538	4.7571	0.9081
T02	35	250	*Ra*_211_ = 2.7422 + 0.0106*t* − 3.46∙10^−5^*t*^2^ − 3∙10^−7^*t*^3^	2.4469	10.7946	4.7571	0.8437
		300	*Ra*_212_ = 1.3088 + 0.0020*t* − 2.8∙10^−5^*t*^2^	2.1788	7.2912	3.8853	0.5486
		350	*Ra*_213_ = 2.4232 + 0.0128*t* − 6.29∙10^−5^*t*^2^ − 9∙10^−8^*t*^3^	2.2010	9.8034	3.5874	0.7278
	45	250	*Ra*_231_ = 2.6268 + 0.008896*t* − 1.9∙10^−5^*t*^2^ − 8∙10^−8^*t*^3^	2.4469	40.0983	4.7571	0.9525
		300	*Ra*_232_ = 3.1537 + 0.0146702*t* − 3.34∙10^−5^*t*^2^	2.5706	5.8886	5.7861	0.7020
		350	*Ra*_233_ = 2.409 + 0.0022*t* − 2.5∙10^−6^*t*^2^	2.3646	5.0613	4.7375	0.5912

**Table 5:** Experimental design and experimental (*T_i_*, *Ra_i_*) and regression model-determined $\left({\hat{T}}_{i},\;{\hat{\mathrm{Ra}}}_{i}\right)$ values.

No	Control Factors		Response Variables							
	*v_c_* (m/min)	*H* (HRC)	Cutting Tool Lifetime *T_i_,* ${\hat{T}}_{i}$ (min)				Surface Roughness ${\mathrm{Ra}}_{i}{,\hat{\mathrm{Ra}}}_{i}$ (μm)			
			*T*_1_	${\hat{T}}_{1}$	*T*_2_	${\hat{T}}_{2}$	*Ra*_1_	${\hat{\mathrm{Ra}}}_{1}$	*Ra*_2_	${\hat{\mathrm{Ra}}}_{2}$
1	250	35	6.67	6.66	5.83	5.80	1.58	1.61	1.85	1.85
2	350	35	4.20	4.14	10.10	10.03	2.40	2.43	1.65	1.65
3	250	45	11.3	11.35	5.33	5.56	0.89	0.86	1.50	1.50
4	350	45	9.20	9.20	8.33	8.40	1.40	1.37	1.40	1.40
5	250	40	9.0	8.95	5.58	5.58	1.24	1.24	1.68	1.68
6	350	40	6.58	6.62	8.92	9.12	1.89	1.90	1.53	1.53
7	300	35	5.33	5.40	7.98	7.92	2.18	2.09	1.75	1.75
8	300	45	10.33	10.28	7.42	6.98	1.15	1.18	1.45	1.45
9	300	40	7.78	7.79	7.25	7.35	1.57	1.63	1.60	1.60

**Table 6:** Regression models and statistical analysis.

Insert	Regression Models	Student’s Criterion	Fisher’s Criterion		Coefficients of Determination	
		*t*_α/2,_	$\hat{F}\geq F_{\alpha ,\nu_{1},\nu_{2}}$		${\hat{R}}_{\mathrm{adj}}^{2}$	${\hat{R}}_{\mathrm{pred}}^{2}$
			$\hat{F}$	$F_{\alpha ,\nu_{1},\nu_{2}}$		
T01	*T*_1_ = 3.0166 − 0.03812*v_c_* + 0.211*H* + 0.0021*H*^2^ + 0.0004*v_c_H*	2.776	2469.59	6.3882	0.9992	0.9971
	$Ra_{1}=-2.7467+0.0347v_{c}-2.667\cdot 10^{-5}v_{c}^{2}-0.0003v_{c}H$	2.5706	182.554	5.409	0.9855	0.9676
T02	*T*_2_ = −9.6736 + 0.0906*v_c_* + 0.004*H*^2^ − 0.0014*v_c_H*	2.5706	111.2501	5.409	0.9764	0.9343
	*Ra*_2_ = 4.240 − 0.0055*v_c_* − 0.0493*H* − 0.0001*H*^2^ + 0.0001*v_c_H*	2.7765	10,276.33	6.3882	0.9998	0.9997

**Table 7:** Analysis of variance results.

Response Variable	Source of Variation	Sum of Squares	Degrees of Freedom (ν)	Mean Square (Variance)	*F*-Value($\hat{F}$)	*p*-Value	Percentage Contribution (%)
Tool life *T*_1_ (min)	*v_c_* (m/min)	8.1436	2	4.0718	314.9661	0.00004	18.56
	*H* (HRC)	35.6782	2	17.8391	1379.9029	0.000002	81.32
	Error (Residual)	0.0517	4	0.0129	-	-	0.12
	Total	43.8735	8	-	-	-	100.00
Surface roughness *Ra*_1_ (µm)	*v_c_* (m/min)	0.66223	2	0.3311	33.6947	0.00314	34.19
	*H* (HRC)	1.2353	2	0.6176	62.8468	0.00095	63.78
	Error (Residual)	0.0393	4	0.0098	-	-	2.03
	Total	1.9369	8	-	-	-	100.00
Tool life *T*_2_ (min)	*v_c_* (m/min)	18.8434	2	9.4217	64.6304	0.00090	90.23
	*H* (HRC)	1.4582	2	0.7291	5.0013	0.08160	6.98
	Error (Residual)	0.5831	4	0.1458	-	-	2.79
	Total	20.8846	9	-	-	-	100.00
Surface roughness *Ra*_2_ (µm)	*v_c_* (m/min)	0.0338	2	0.0169	26.8850	0.0048	19.71
	*H* (HRC)	1.1350	2	0.0675	107.5398	0.0003	78.83
	Error (Residual)	0.0025	4	0.0006	-	-	1.46
	Total	20.8846	9	-	-	-	100.00

Note: The theoretical critical value of the *F*-distribution at a 95% confidence level is $F_{\alpha ,\nu_{1},\nu_{2}}=6.9443$.

**Table 8:** Effect of cutting conditions on performance criteria.

Insert	Change in Performance Criteria (%)			
	*v_c_*_min_ = 250 m/min *v_c_*_max_ = 350 m/min *H*_m_ = 40 HRC		*H*_min_ = 35 HRC *H*_max_ = 45 HRC *v_cm_* = 300 m/min	
	*ΔT*	*ΔRa*	*ΔT*	*ΔRa*
T01	26.9	26.2	48.4	47.2
T02	26.2	19.0	7.01	17.14

**Table 9:** Limits of variation in the response variables over the range of cutting conditions investigated.

Insert	*v_c_*_min_ = 250 m/min *H*_min_ = 35 HRC		*v_c_*_max_ = 350 m/min *H*_max_ = 45 HRC	
	*T* (min)	*Ra* (µm)	*T* (min)	*Ra* (µm)
T01	6.67	1.58	9.2	1.4
T02	5.83	1.85	8.33	1.4

**Table 10:** Predicted optimal conditions for turning with ZTA and mixed-ceramic inserts.

Insert	Optimum Turning Conditions		Response Variables	
	$v_{c}^{*}$ (m/min)	H^*^ (HRC))	*T* (min)	*Ra* (µm)
T01	250.0	45.0	11.349	0.8585
T02	349.85	35.96	9.839	1.627

**Table 11:** Design of the experiment and generalized utility functions.

No	Controlfactors		ZTA Ceramic Tools		Mixed-Alumina Ceramic Tools		ZTA Ceramic Tools			Mixed-Alumina Ceramic Tools		
	*v_c_* (m/min)	*H* (HRC)	η_T1_	η_Ra1_	η_T2_	η_Ra2_	Φ_11_	Φ_12_	Φ_13_	Φ_21_	Φ_22_	Φ_23_
1	250	35	0.348	0.543	0.230	0.364	0.446	0.407	0.485	0.297	0.270	0.324
2	350	35	0.000	0.000	0.831	0.500	0.000	0.000	0.000	0.666	0.732	0.599
3	250	45	1.000	1.000	0.159	0.596	1.000	1.000	1.000	0.378	0.290	0.465
4	350	45	0.704	0.662	0.582	0.662	0.683	0.691	0.675	0.622	0.613	0.638
5	250	40	0.676	0.768	0.194	0.477	0.722	0.704	0.740	0.336	0.279	0.392
6	350	40	0.335	0.338	0.665	0.576	0.337	0.336	0.337	0.621	0.638	0.603
7	300	35	0.159	0.146	0.532	0.430	0.153	0.155	0.150	0.481	0.501	0.461
8	300	45	0.863	0.828	0.453	0.629	0.846	0.853	0.839	0.541	0.506	0.576
9	300	40	0.504	0.550	0.430	0.529	0.527	0.518	0.536	0.480	0.460	0.499

**Table 12:** Regression models of the generalized utility function and statistical characteristics of the models.

Regression Models	Student’s Criterion	Fisher’s Criterion		Coefficient of Determination	
	*t*_α/2,_	$\hat{F}\geq F_{\alpha ,\nu_{1},\nu_{2}}$		${\hat{R}}^{2}$	${\hat{R}}_{\mathrm{pred}}^{2}$
		$\hat{F}$	$F_{\alpha ,\nu_{1},\nu_{2}}$		
Zirconia-toughened alumina ceramic tools					
Φ_11_ = 1.6718 − 0.0123*v_c_* + 0.00021*v_c_H*	2.4469	487.378	5.1433	0.9939	0.9828
Φ_12_ = 0.1355 − 0.0075*v_c_* + 0.0367*H* + 9.8 × 10^−5^*v_c_H*	2.5707	863.033	5.4095	0.9981	0.9910
Φ_13_ = 1.7421 − 0.0124*v_c_* + 0.00021*v_c_H*	2.4469	380.169	5.1433	0.9922	0.9809
Mixed-alumina ceramic tools					
Φ_21_ = −1.2548 + 0.0082*v_c_* + 0.0052*H*^2^ − 0.00013*v_c_H*	2.5706	174.935	5.4095	0.9906	0.9519
Φ_22_ = −1.5085 + 0.0098*v_c_* + 0.00052*H*^2^ − 0.00015*v_c_H*	2.5706	137.304	5.4095	0.9880	0.9442
Φ_23_ = −0.9692 + 0.0063*v_c_* + 0.00051*H*^2^ − 0.00015*v_c_H*	2.5706	326.632	5.4095	0.9949	0.9712

**Table 13:** Predicted optimal conditions and response variables for turning with inserts made of ZTA and mixed aluminum ceramic.

Insert	Optimal Conditions		Response Variables		Generalized Utility Functions					
	$V_{c}^{*}$ (m/min)	H^*^ (HRC)	*T* (min)	*Ra* (µm)	Φ_11_	Φ_12_	Φ_13_	Φ_21_	Φ_22_	Φ_23_
T01	250.0	45	11.3	0.89	0.9812	-	-	-	-	-
					-	1.0047	-	-	-	-
					-	-	0.9911	-	-	-
T02	350.0	35	10.1	1.65	-	-	-	0.6613	-	-
					-	-	-	-	0.7241	-
		45	8.33	1.40	-	-	-	-	-	0.6440

**Table 14:** Predicted optimal conditions that ensure a combination of the maximum values of the generalized utility functions Φ_1*r*_ and Φ_2*r*_.

Optimal Conditions		Performance Criteria				Generalized Utility Functions					
$v_{c}^{*}$ (m/min)	H^*^ (HRC)	*T*_1_ (min)	*Ra*_1_ (µm)	*T*_2_ (min)	*Ra*_2_ (µm)	Φ_11_	Φ_12_	Φ_13_	Φ_21_	Φ_22_	Φ_23_
344.3	45	9.37	1.36	8.25	1.41	0.7207	-	-	0.6160	-	-
347.9	45	9.30	1.37	8.35	1.40	-	0.6989	-	-	0.6125	-
344.6	45	8.37	1.36	8.26	1.41	-	-	0.7071	-	-	0.6348

## Data Availability

The original contributions presented in this study are included in the article. Further inquiries can be directed to the corresponding author.
